# High efficacy *in vitro* selection procedure for generating transgenic parasites of *Plasmodium berghei* using an antibiotic toxic to rodent hosts

**DOI:** 10.1038/s41598-017-04244-0

**Published:** 2017-06-21

**Authors:** Akira Soga, Hironori Bando, Mami Ko-ketsu, Hirono Masuda-Suganuma, Shin-ichiro Kawazu, Shinya Fukumoto

**Affiliations:** 1 National Research Center for Protozoan Diseases, Obihiro University of Agriculture and Veterinary Medicine, Inada-cho, Obihiro, Hokkaido 080-8555 Japan; 2 Department of immunoparasitology, Research Institute for Microbial Disease, Osaka University, Yamada-oka, Suita, Osaka 565-0871 Japan

## Abstract

The malaria parasite *Plasmodium berghei* is one of the main rodent malaria models. A shortcoming of this model parasite is its low flexibility in genetic manipulation. As this parasite cannot be continuously propagated in cell cultures, *in vivo* drug selection procedures are necessary to isolate genetic mutants. Drugs harmful to rodents therefore cannot be used for drug selection, which restricts the range of genetic manipulation. In this study, we addressed this problem by establishing a novel *in vitro* culture drug selection method, which we used in combination with other established methods to successfully isolate genetically manipulated parasites. The target mutants were enriched to the desired level within two weeks. We show that our system can also be used for sequential genetic manipulation of parasites carrying the traditionally used selection markers, demonstrate the procedure’s versatility, and show its use in isolating specific genetically manipulated parasites. This novel *in vitro* selection method increases the number of available selection markers, allowing more extensive genetic manipulation in malaria parasite research.

## Introduction

Malaria is a global life-threatening disease caused by protozoan parasites of the genus *Plasmodium*. It has been estimated that half of the world’s population is at risk of infection, and the disease causes approximately 500,000 deaths annually^1^. Consequently, high priorities are being placed on developing novel strategies to control malaria.

*Plasmodium berghei* (*P. berghei*) is used as the main rodent malaria model^2^. *P. berghei* is harmless to humans, its full lifecycle can be completed in a laboratory (including the stages occurring in the mosquito and the host’s liver), and a reverse genetics approach has been established for studying it^3, 4^. The evolutionary distance between the *P. berghei* clade and either *P. falciparum* or *P. vivax* is of the same order of magnitude as that between *P. falciparum* and *P. vivax* ^2, 5^.

Although *P. berghei* has proven a useful model parasite, its use is circumscribed by the limited number of drug selection markers that can be effectively employed with it, reducing the scope of genetic manipulation. *In vivo* drug selection procedures using rodents are required for the isolation of genetically manipulated *P. berghei*, as the parasite cannot be continuously propagated through *in vitro* cultures. Since this rules out drugs that are harmful to mammalian hosts, the use of popular mammalian drug selection marker genes is largely precluded^3^. This leads to difficulties in running sequential genetic manipulation experiments, such as phenotype rescue experiments using gene knockout (KO) parasites or the generation of multiple KO parasites^6–8^. These circumstances have been impeding malaria research.

Three positive selection markers are available for *P. berghei*: 1) *Toxoplasma gondii* reductase-thymidylate synthase (*tgdhfr-ts*); 2) pyrimethamine-resistant *P. berghei dhfr-ts* (*pbdhfr-ts*); and 3) human *dhfr* (*hdhfr*)^2^. All three markers confer resistance to antifolate pyrimethamine, but only *hdhfr* confers resistance to WR99210^6^ and can thus be used as a secondary marker in sequential manipulation experiments. However, sequential manipulation is challenging because *hdhfr* only confers slight resistance to WR99210^6^, and only two reports describe sequential gene disruptions using these markers^2, 9, 10^. A marker recycling method using negative selection was developed to address this issue^7, 11, 12^, but did not prove to be an effective solution.

In this study, we developed a novel selection method to overcome the genetic manipulation problems in *P. berghei* models. We focused on the puromycin-*N*-acetyltransferase (*pac)*-puromycin system, which is commonly used in mammalian cells^13, 14^. *pac* can be isolated from *Streptomyces alboniger* and shows resistance to the aminonucleoside antibiotic, puromycin^15^. A previous study reported that *pac* might be used as a selection marker for blood stage long-term *in vitro* cultures of *P. falciparum*, the causative agent of tropical malaria in humans^15^. As puromycin is severely toxic to rodents and thus cannot be used for *P. berghei in vivo*, we developed a *pac*-puromycin system based on a novel short-term *in vitro* culture method.

## Results

### Expression of *pac* confers resistance to puromycin

**Figure 1:**
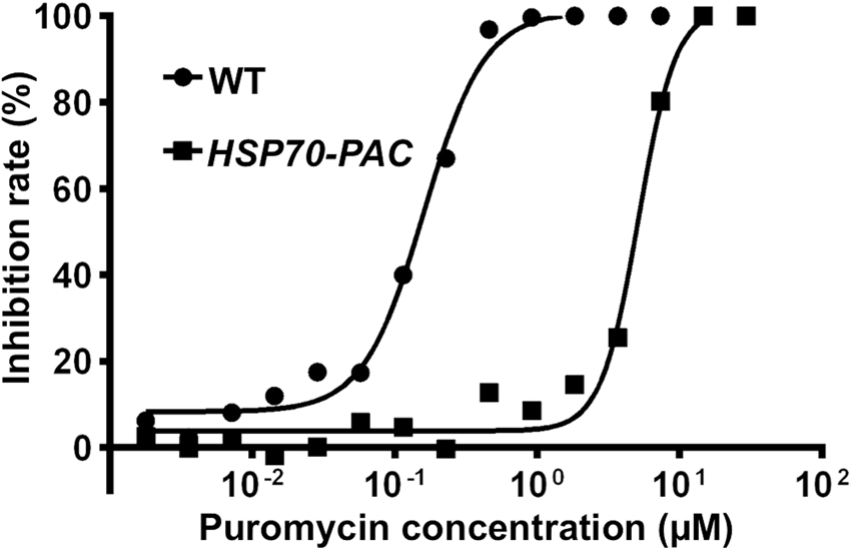
Puromycin resistance assay of wild type and *pac*-transfected parasites. Growth inhibition of wild type (WT) and pXL/hdhfr-pac-egfp (Fig. 2a)-transfected parasites (*HSP70-PAC*) was assessed by determining schizont development at a range of puromycin concentrations.

To determine whether *pac* can confer puromycin resistance to *P. berghei*, parasites transfected with pXL/hdhfr-pac-egfp carrying *pac* under the control of *hsp70* promoters (*HSP70-PAC*) were obtained using *in vivo* pyrimethamine selection. IC_50_ values of wild type and *HSP70-PAC* were 0.17 ± 0.06 µM and 5.69 ± 0.70 µM (mean ± SD), respectively (Fig. 1). IC_90_ values of wild type and the mutant parasites were 0.55 ± 0.14 µM and 9.39 ± 1.07 µM (mean ± SD), respectively (Fig. 1).

We identified a suitable puromycin concentration for drug selection by selecting pXL/hdhfr-pac-egfp-transfected parasites at various puromycin concentrations. The empirical optimal concentration was 1.0 µg/ml (1.8 µM) and the target mutant was not obtained at concentrations above 1.5 µg/ml.

### Generation of transgenic parasites using *pac* markers through *piggyBac* transposon

**Figure 2:**
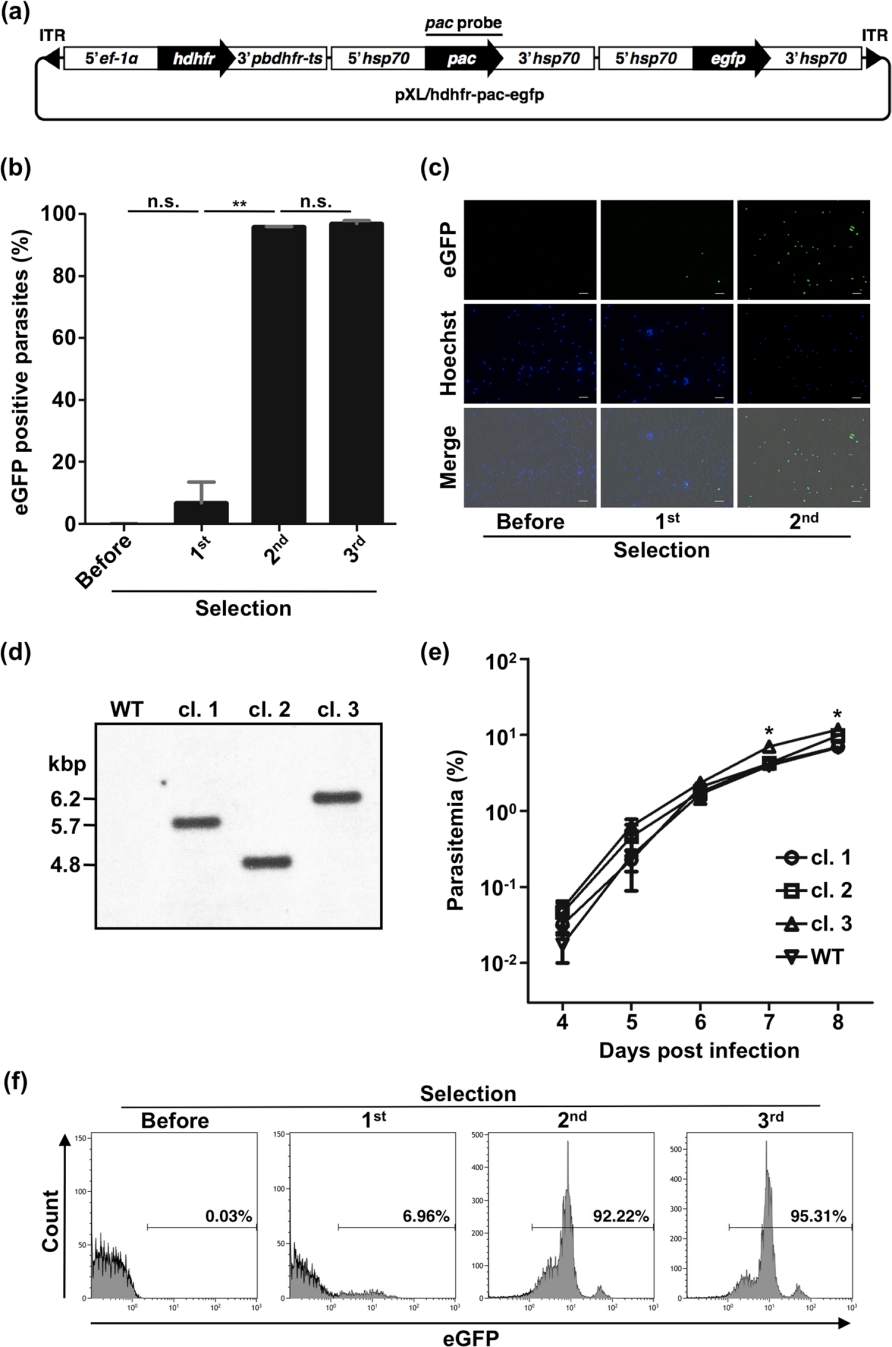
Generation of mutants using the *pac* marker and *piggyBac* transposon. (**a**) Schematic diagram of the *piggyBac* transposon vector containing *hdhfr*, *pac* and *egfp* expression cassettes (pXL/hdhfr-pac-egfp). This plasmid was also used in the experiment of Figs 1 and 6. ITR: inverted terminal repeat. (**b**) eGFP-positive parasite ratio after each puromycin selection. The ratio was analyzed using fluorescence microscopy. Each bar represents the mean ± SD of three independent experiments. **p < 0.01, n.s.: not significant (paired *t*-tests). (**c**) Fluorescence images of parasites after each selection. Parasites were stained with Hoechst 33342. The scale bar represents 10 µm. (**d**) Southern blot analysis of three mutant clones. Genomic DNA was digested using *Eco* RV and hybridized with a *pac* probe. The predicted sizes of restriction fragments are shown. WT: wild type. cl.: clone. Full-length blot image is presented in Supplementary Figure S1. (**e**) Growth assay of three clones. cl.: clone. WT: wild type. *p < 0.05 (two-tailed unpaired *t*-tests). (**f**) Flow cytometry analysis of eGFP positive mutant ratio after each puromycin selection. Numbers above bracketed lines indicate percent parasites with eGFP expression.

To determine whether *pac* can be used as a positive selection marker for genetically manipulated parasites, we used it to select pXL/hdhfr-pac-egfp-transfected parasites (Fig. 2a). Three selections were performed after transfection: the first after two days, the second after 8–9 days, and the third after 13–14 days. After the second selection, over 95% of parasites were expressing enhanced green fluorescent protein (eGFP) (Fig. 2b,c). The target mutant ratio increased significantly from first to second selections. There was no significant difference between second and third selections (Fig. 2b). A typical transfectant line was also analyzed using flow cytometry. Over 95% of parasites were expressing eGFP after the third selection (Fig. 2f). Three clones were obtained and analyzed using Southern blot analysis (Fig. 2d) to confirm genomic integration. The fragments of integrated *pac* cassettes (4.8, 5.7, 6.2 kbp) were detected with a *pac* probe. We did not detect a signal corresponding to plasmid size. Plasmid rescues on parasite DNA extracted from these clones confirmed the absence of episomally maintained plasmids. Sequence analyses of inverse PCR products showed that these three clones had single copy insertions in unique loci (Table 1). To determine the inhibitory effect of Pac expression on parasite growth, the growth of the three *pac* clones was analyzed. Clones 1 and 2 showed no significant difference from wild type parasites, but clone 3 exhibited significantly higher parasitemia at seven and eight days post infection (Fig. 2e). These results demonstrate that *pac* represents a valid selection marker in *P. berghei*.

**Table 1:** *piggyBac* transposon insertion sites.

Clone	Locus	Insertion site
1	Chromosome 13, 136517–136518	TTATTCTA**TTAA**-*piggyBac*-**TTAA**TATTTACA
2	Chromosome 13, 2294432–2294433	AAATAATT**TTAA**-*piggyBac*-**TTAA**AAAAGAAA
3	Chromosome 14, 1045361–1045362	TAATATTG**TTAA**-*piggyBac*-**TTAA**TAAAAATG

### Generation of transgenic parasites using *pac* through double homologous recombination

**Figure 3:**
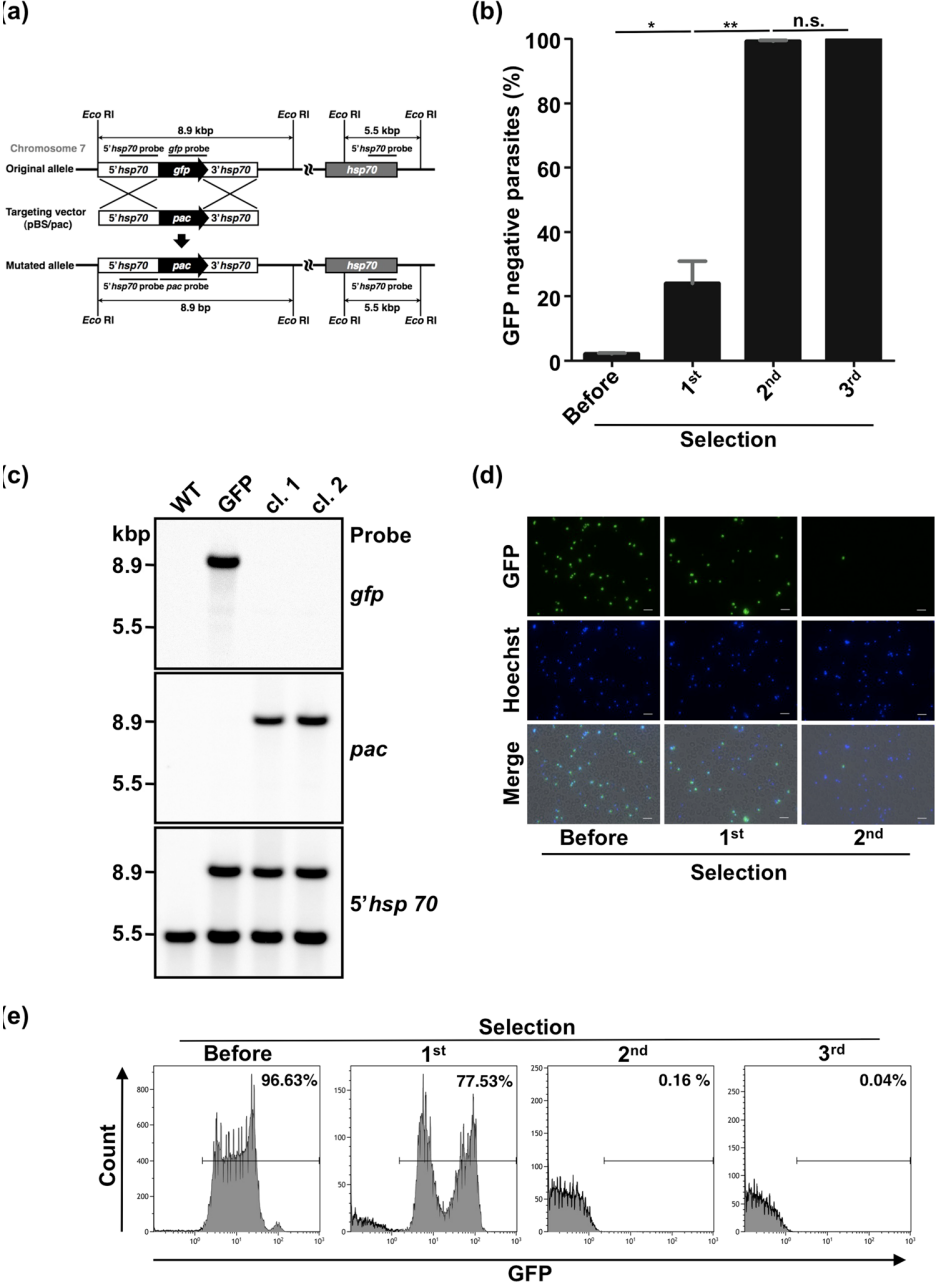
Generation of mutants using the *pac* marker by double-crossover homologous recombination. (**a**) Schematic diagram showing the targeting of *gfp* by *pac* through double crossover homologous recombination. (**b**) GFP negative parasite ratio after each puromycin selection. The ratio was analyzed using fluorescence microscopy. Each bar represents the mean ± SD of three independent experiments. **p < 0.01, *p < 0.05, n.s.: not significant (paired *t*-tests). (**c**) Southern blot analyses of two independent clones. Genomic DNA was digested with *Eco* RI and hybridized with *gfp*, *pac*, and 5′*hsp70* probes. The predicted sizes of restriction fragments are shown. WT: wild type, GFP: GFP-expressing parasite (parental line), cl.: clone. Full-length blot images are presented in Supplementary Figure S2. (**d**) Fluorescence images of parasites after each selection. Parasites were stained with Hoechst 33342. The scale bar represents 10 µm. (**e**) Flow cytometry analysis of GFP positive mutant ratio after each puromycin selection. Numbers above bracketed lines indicate percent parasites with GFP expression.

We investigated whether *pac* can be used for gene targeting by assembling constructs that contained *pac* under the control of an *hsp70* promoter and terminator (Fig. 3a). We used a GFP-expressing parasite^16^ under the control of an *hsp70* promoter and terminator as a parental strain for transfection. As shown in Fig. 3a, the *gfp* expression cassette would be replaced by the *pac* expression cassette after the homologous recombination event, and GFP expression would be lost.

Three selections were performed after transfection: the first after 2 days, the second after 8 days, and the third after 12 days. After the second selection, more than 99% of parasites were GFP-negative (Fig. 3b,d). The mutant ratio increased significantly from first to second selections, but there was no significant difference between second and third selections (Fig. 3b). A typical transfectant line was also analyzed using flow cytometry. More than 99% of parasites were GFP-negative after the second selection (Fig. 3e). From two of these experiments we obtained two independent clones, which were analyzed using Southern blot analyses (Fig. 3c). The 8.9-kbp fragment containing the *gfp* cassette was only detected in the parental GFP-expressing parasite with the *gfp* probe. The identical 8.9-kbp signals were detected in both clones, but not in the wild type, with the *pac* probe and the 5′*hsp70* probe. Based on these results, the *in vitro pac*-puromycin system is suitable for gene targeting as well as random insertion by transposon.

### Sequential genetic manipulation of *pac* to *pbdhfr-ts* parasites

**Figure 4:**
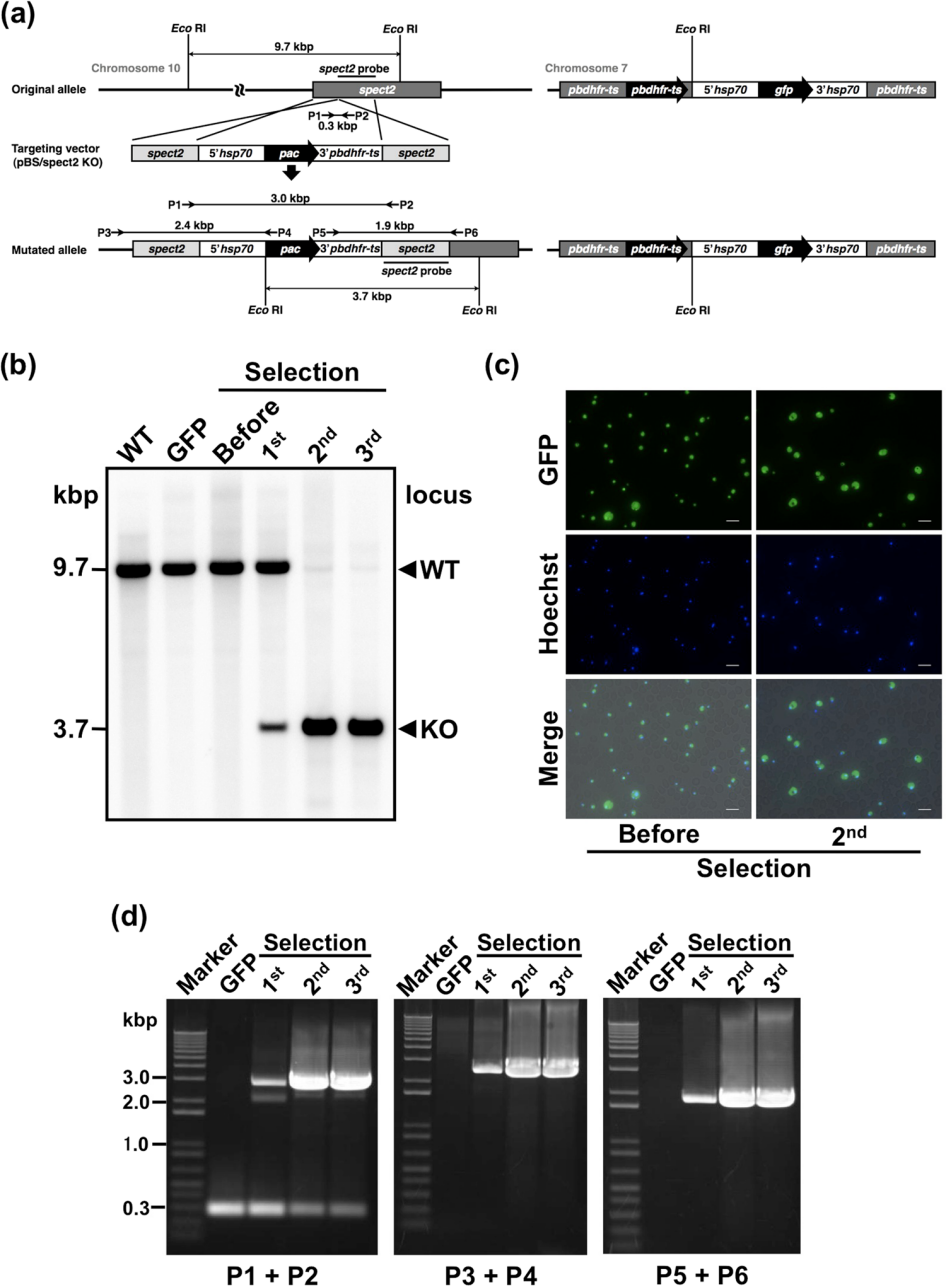
Generation of double mutants using *pac* and *pbdhfr-ts* markers. (**a**) Schematic diagram showing the targeting of *spect2*. *pac* was inserted into the *spect2* locus via double crossover homologous recombination. *pbdhfr-ts* was used to obtain the parent GFP transgenic parasite by *in vivo* pyrimethamine selection^16^. (**b**) Southern blot analysis showing the ratio of *spect2* KO mutants after each selection. Genomic DNA was digested using *Eco* RI and hybridized with a *spect2* probe. WT: wild type, KO: *spect2* KO. (**c**) Fluorescence images of parental GFP-expressing parasites and a *spect2* KO mutant parasite population after the second selection containing *pac* and *pbdhfr-ts* markers. Parasites were stained with Hoechst 33342. The scale bar represents 10 µm. Before: parental GFP-expressing mutant. (**d**) PCR analysis of genomic DNA isolated from mutant parasites recovered after each selection. Location of PCR primers and the expected products size are shown in (**a**). Confirmation of the predicted recombination events was achieved with primer combinations specific for 5′ (P3 + P4) or 3′ integration (P5 + P6). An additional primer combination (P1 + P2) was used to assess integration of the *pac* cassette. The primer sequences can be found as Supplementary Table S1. P: primer.

We demonstrated sequential genetic manipulation using *pac* and *pbdhfr-ts* markers for gene targeting. To this end, we assembled a targeting vector composed of a *pac* cassette flanked by sporozoite microneme proteins essential for the cell traversal 2 (*spect2*, PBANKA_1006300)^17^ gene sequence (Fig. 4a). A GFP-expressing parasite^16^ containing *pbdhfr-ts* marker was used as the parental strain for transfection. The parasite was transfected with the targeting construct, after which puromycin selection was applied. The ratio of the target mutant was determined using genomic Southern blot analysis (Fig. 4b). KO locus signals (3.7 kbp) appeared after the first selection but became dominant after the second selection. We confirmed that GFP was continuously expressed from before transfection to after third selection (Fig. 4c). The KO of *spect2* was confirmed by PCR (Fig. 4d). These results show that the *in vitro pac*-puromycin system can be used in combination with traditional *pbdhfr-ts* markers to establish double mutant parasites.

### Generation of mutants using the *pac::egfp* fusion marker

**Figure 5:**
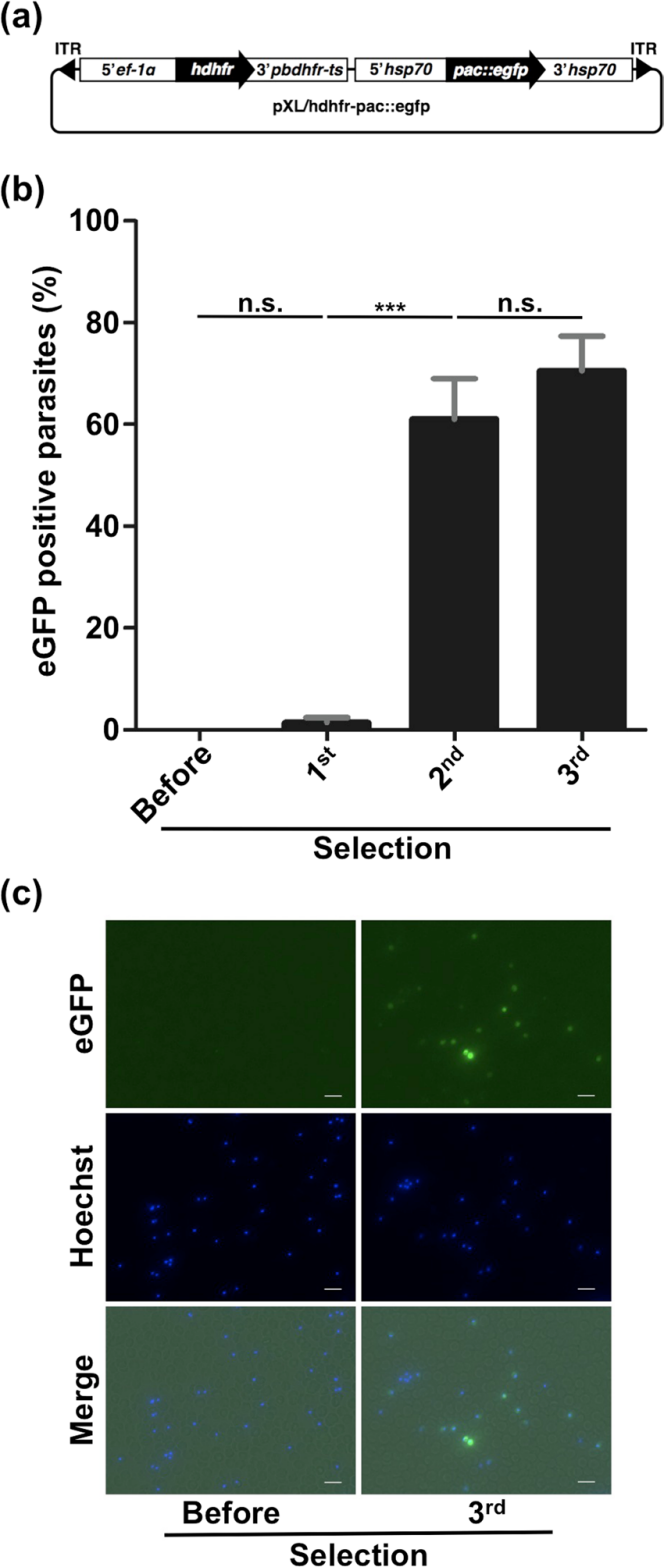
Generation of mutants using the *pac::egfp* fusion marker. (**a**) Schematic diagram of *piggyBac* transposon vector containing the *pac::egfp* fusion marker (pXL/hdhfr-pac::egfp). ITR: inverted terminal repeat. (**b**) eGFP-positive parasite ratios after each puromycin selection. Ratios were analyzed using fluorescence microscopy. Each bar represents the mean ± SD of four independent experiments. ***p < 0.001, n.s.: not significant (paired *t*-tests). (**c**) Fluorescence images of parasites before selection and after the third selection containing the *pac::egfp* fusion marker. Parasites were stained with Hoechst 33342. The scale bar represents 10 µm.

We investigated whether the *pac::egfp* fusion gene can be used as a marker by assembling a *piggyBac* transposon vector that contained the fusion gene under the control of an *hsp70* promoter and terminator (Fig. 5a). To confirm that *pac::egfp* functionally expressed eGFP, *in vivo* pyrimethamine selection of pXL/hdhfr-pac::egfp transfected parasites was performed prior to *in vitro* puromycin selection. The expression of eGFP was confirmed by fluorescence microscopy.

After transfection of pXL/hdhfr-pac::egfp, *in vitro* puromycin selection was conducted three times. Fluorescence analyses showed that more than 60% of parasites expressed *egfp* after the second selection (Fig. 5b). The mutant ratio increased significantly between first and second selections, but there was no significant difference between second and third selections (Fig. 5b). Fluorescence microscopy confirmed the expression of the eGFP signal (Fig. 5c). Based on these results, *pac::egfp* is suitable as a dual marker for fluorescence and drug selection.

### Application of the *in vitro* selection method to the *hdhfr*-WR99210 system

**Figure 6:**
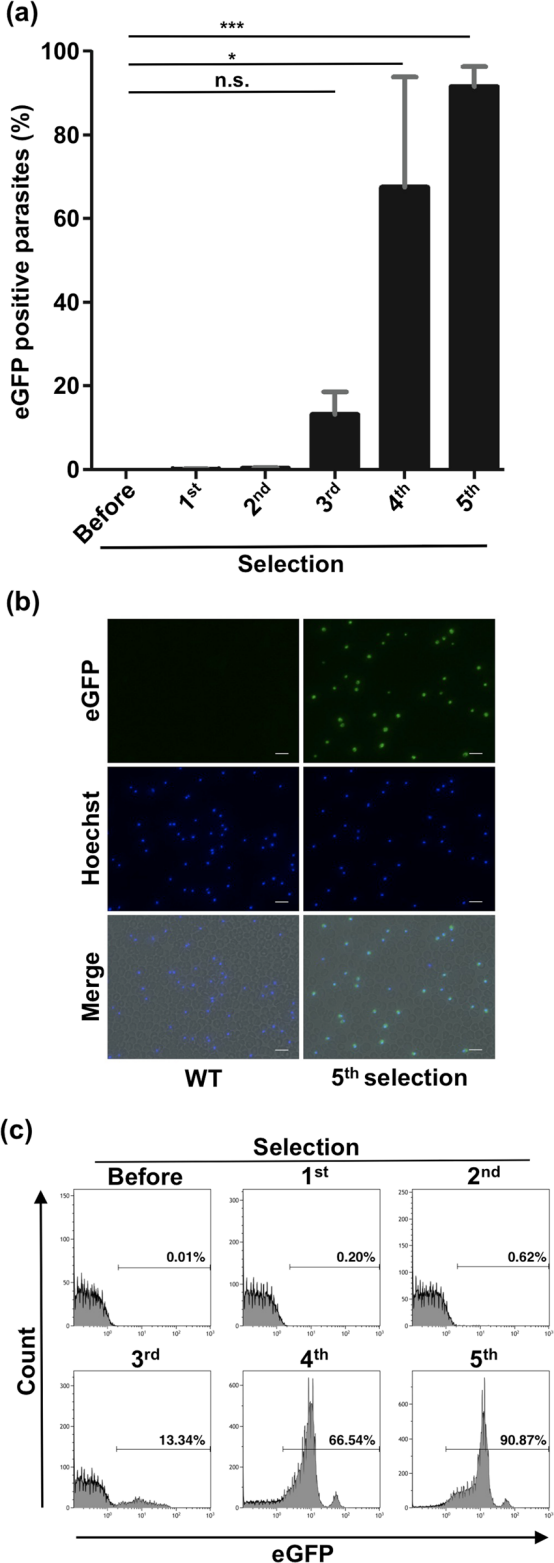
Application of the *hdhfr*-WR99210 system to *in vitro* selection. (**a**) eGFP-positive parasite ratio transfected with pXL/hdhfr-pac-egfp (Fig. 2a) after each WR992210 selection, analyzed using fluorescence microscopy. Each bar represents the mean ± SD of three independent experiments. *p < 0.05, ***p < 0.001, n.s.: not significant (paired *t*-tests). (**b**) Fluorescence images of WT and parasites after the fifth selection containing the *hdhfr* marker. Parasites were stained with Hoechst 33342. WT: wild type. The scale bar represents 10 µm. (**c**) Flow cytometry analysis of eGFP positive mutant ratio after each WR99210 selection. Numbers above bracketed lines indicate percent parasites with eGFP expression.

To examine the universality of our *in vitro* selection method, we used the *hdhfr*-WR99210 system as a model. Preliminary experiments showed that the optimal WR99210 concentration for selection was 6.25 ng/ml (1.6 × 10^−2^ µM). A wild type parasite was transfected with pXL/hdhfr-pac-egfp, after which WR99210 selection was performed. Proportions of eGFP-positive parasites increased significantly up to the fourth and fifth selections, and the mutant ratio reached more than 90% after the fifth selection (Fig. 6a,b). A typical transfectant line was also analyzed using flow cytometry. Over 90% of parasites were expressing eGFP after the fifth selection (Fig. 6c). These results show that the employed *in vitro* selection method may be suitable for application in drug selection systems other than the *pac*-puromycin system.

## Discussion

Reverse genetics has been a powerful approach in studying the biology of malaria parasites^3, 4^. However, the limited number of selection markers has hampered the development of the method^6, 7^. To provide further selection markers, we developed a *pac* marker in *P. berghei* based on a novel *in vitro* selection method.

We established this method by combining repeated short-term *in vitro* cultures with parasite recovery *in vivo*. The *pac* marker conferred sufficient resistance to the *P. berghei* parasite for puromycin selection. Its resistance made it possible to isolate the mutant parasites using *piggyBac* transposon mutagenesis and gene targeting by homologous recombination. We showed that *pac::egfp* was also an effective drug selection marker and might constitute a useful tool for imaging experiments. Blood stage parasite growth analyses of Pac-expressing clones indicated that our *pac* marker cassette did not inhibit parasite growth. While currently available positive selection markers for this parasite only confer resistance to antifolates^2^, *pac* is independent of antifolate pathways^15^. Thus, the *pac*-puromycin system can be used in conjunction with the traditional antifolate resistant markers (*hdhfr* and *pbdhfr-ts*). The small size of *pac* (600 bp) is one of its advantages; in *Plasmodium*, assembling targeting constructs causes a transfection bottleneck because large-sized constructs often result in instability in *E. coli* caused by the AT-rich *Plasmodium* genome.

We confirmed the universality of this *in vitro* selection method by using the traditional *hdhfr*-WR99210 system as a model. The results indicate that our *in vitro* method has potential for use in other drug selection systems already established for *P. falciparum* ^18^. In addition, this system could also be used for negative selection *in vitro*, to recycle markers. A further benefit of our method is its relative cheapness, as WR99210 is approximately 100 times more expensive than pyrimethamine (based on Sigma Aldrich prices). The new system significantly reduces the amount of WR99210 required (0.001%) relative to the conventional *in vivo* method.

Our method can enrich mutant parasites by more than 90% within 2 weeks after transfection, using 2 mice. This level of efficacy has great advantages for cloning mutant parasites, and may reduce the number of mice required to obtain the desired production. Traditional cloning procedures involve using the *in vivo* limiting dilution method^1^. In our experience, the percentage of target KO parasites obtained when using the conventional selection method is around 10–20%, so double limited dilution procedures and at least 20 mice are required to isolate a single cloned parasite. Our *in vitro pac*-puromycin system, however, allows us to isolate a clone using fewer than five mice. This reduction may save significant expense, time, and labor in rearing mice, and has obvious animal welfare benefits. Furthermore, the method allows the ready generation of several mutants in parallel. In some cases, the drug-selected parasites can be used directly without cloning or flow-sorting steps^2^.

A further advantage of this system is its flexible application for sequential manipulation to mutant parasite generated using traditional antifolate-resistant markers. While various strategies have successfully used negative selection in the *P. berghei* model^7, 12^, the disadvantage of the traditional marker recycling method is its complexity and the time requirement of several months for the isolation of a double mutant parasite^7, 19^. The “gene out marker out” (GOMO) strategy was developed to address this problem^12^. However, this method is inapplicable to parasites already carrying antifolate resistant markers as it requires positive selection using antifolates. Our *in vitro pac*-puromycin system can readily be used in sequential manipulation experiments (for example when employing gene complementation methods) with the mutant parasites established in past malaria research, using any type of selection method including traditional antifolate markers. In our experiment, the target cloned parasite was obtained within 3 weeks using less than 10 mice.

In summary, we succeeded in establishing a novel *in vitro* selection method using the *pac* marker in *P. berghei*. This *in vitro pac*-puromycin selection system exhibited high selection efficacy relative to the conventional *in vivo* method. The method should allow the generation of multiple mutants with greater flexibility and help develop our in-depth understanding of malaria parasite biology.

## Methods

### Ethics statement

This study was carried out in strict accordance with the recommendations in the Guide for Laboratory Animals of the Obihiro University of Agriculture and Veterinary Medicine. The protocol was approved by the Committee of Animal Experiments of the Obihiro University of Agriculture and Veterinary Medicine (permit number 28–91).

### Experimental animals and parasites

ICR and BALB/c (five-week old) mice were obtained from CLEA (Tokyo, Japan). We used the *P. berghei* ANKA strain (obtained from Dr M. Torii, Ehime University) and a strain constitutively expressing GFP^16^ (obtained from Dr M. Yuda, Mie University).

### Plasmid construct

Elements of pXL/hdhfr-pac-egfp and pXL/hdhfr-pac::egfp plasmids were sequentially ligated into a pXL-BacII-DHFR (obtained from the BEI Resources Repository, MRA-916, deposited by John Adams) plasmid backbone^20, 21^. Firstly, the *hdhfr* expression cassette was excised from pXL-BacII-DHFR, namely pXL-BacII-DHFR (-). Next, *hdhfr*, under the control of *elongation factor 1 alpha* (*ef-1α*) promoter and *pbdhfr-ts* terminator, was cloned into pXL-BacII-DHFR (-). Then, for pXL/hdhfr-pac-egfp, *egfp* was excised from pCX-EGFP Vector^22^ under the control of an *hsp70* promoter and terminator and was then cloned into the plasmid pXL-BacII-DHFR (-) containing the *hdhfr* expression cassette. *pac* was then excised using a pMXs-Puro retroviral vector (Cell Biolabs, San Diego, CA, USA) under the control of an *hsp70* promoter and terminator, and cloned in the pXL-BacII-DHFR (-) containing the *hdhfr* and *egfp* expression cassettes. For pXL/hdhfr-pac::egfp, *pac* was cloned into a pEFP-N1 vector (Clontech, Palo Alto, CA, USA), then the resulting *pac::egfp* fusion gene was cloned into the plasmid pXL-BacII-DHFR (-) containing the *hdhfr* expression cassette, under the control of another *hsp70* promoter and terminator. The promoter and terminators were excised from a Y2 plasmid (obtained from Dr M. Yuda, Mie University).

The transposase expression vector EGF-pgT contained a transposase gene excised from pHTH (obtained from the BEI Resources Repository, MRA-912, deposited by John Adams), under the control of an *ef-1α* promoter and *hsp70* terminator, cloned into a pBluescript (pBS) vector^20^.

Elements of pBS/spect2 KO were generated into a pBS backbone. The *pac* expression cassette was flanked by 5′ and 3′ sequences of *spect2* (pBS/spect2 KO) amplified by PCR (Table S1).

### Parasite transfection and drug selection *in vitro*

Parasite transfection experiments were run following standard protocols^23^. Briefly, about 1 ml of infected blood (1.0–3.0% parasitemia) collected from an ICR mouse by heart puncture was cultured in 50 ml of RPMI 1640 medium (cat no. 23400–021, Thermo Fisher Scientific, MA, USA,) supplemented with NaHCO_3_ (0.85 g/l), neomycin sulfate (50 mg/l), and 20% fetal bovine serum for schizonts collection. Schizonts were purified by Nycodenz density gradient centrifugation^24^. Obtained schizonts were sufficient for at least 5 independent transfections. Transfection was performed with a Nucleofector 2b device (Lonza, Basel, Switzerland) using a T Cell Nuclofector Kit (Lonza) under the U-33 program. All *in vitro* selection procedures were performed separately from the *in vivo* drug selection using pyrimethamine or WR99210. All *in vitro* culture and drug selection procedures were performed without shaking, and started at a time between 13:00 h and 15:00 h.

During the *piggyBac* experiment, 5 μg of target plasmid was co-transfected with 5 μg of transposase plasmid EGF-pgT. In the double crossover homologous recombination experiment, 10 μg of linearized plasmid was used in each transfection. After transfection, the parasites were immediately injected intravenously into a naive ICR mouse. The first drug selection was performed about 1–2 days post-injection when parasitemia reached 0.5–3.0%.

In *piggyBac* experiments, about 7 μl of infected tail blood were collected in 0.5 ml of culture medium (described above) with 2 μl of heparin solution (143 units/ml), and centrifuged at 500 × *g* for 5 min at room temperature (RT). The supernatant was discarded and the parasites were resuspended in 0.5 ml of culture medium. The suspension (450 µl) was put into a 24-well plate (Thermo Fisher Scientific), and either 50 µl of either puromycin solution (10 µg/ml, diluted by culture medium; Wako, Osaka, Japan; stock: 50 mg/ml in distilled water) to a final concentration of 1.0 µg/ml (1.8 µM), or WR99210 solution (62.5 ng/ml, diluted by culture medium; Jacobus Pharmaceuticals; stock: 5 mg/ml in DMSO) to a final concentration of 6.25 ng/ml (1.6 × 10^–2^ µM) was added. The parasites were incubated for 20 h (36.5 °C, 5% CO_2_, 5% O_2_, 90% N_2_). After incubation, the parasites were separated by centrifuge at 500 × *g* for 5 min at RT, and resuspended in 100 μl of PBS. They were then injected intravenously into a naive ICR mouse. When parasitemia reached 0.5–3.0% (about 5 days post-injection), the same selection procedure was repeated for the 2^nd^ and 3^rd^ selection using about 7 μl of tail blood. Cloning of parasites was done by *in vivo* limiting dilution method using male BALB/c mice.

In the first selection of double crossover homologous recombination experiments, 200 μl of infected blood was collected in 5 ml of culture medium by heart puncture under anesthesia, using a syringe containing heparin solution. After centrifugation at 500 × *g* for 8 min at RT, the supernatant was discarded. The blood was resuspended in 10.8 ml of culture medium. The suspension was put into a 25 cm^2^ flask (Thermo Fisher Scientific) with 1.2 ml of puromycin (10 µg/ml, diluted by culture medium) solution to make a final concentration of 1.0 µg/ml and a total volume of 12 ml. This suspension was then incubated for 20 h. After drug selection, the sample was centrifuged at 500 × *g* for 8 min at RT and resuspended in 100 μl of PBS. The suspension was injected intravenously into a naive ICR mouse. When parasitemia reached 0.5–3.0% (about 5 days post-injection), the selection procedure was repeated. In the 2^nd^ and 3^rd^ selection, about 7 μl of tail blood in 0.5 ml culture medium was used as described above for the *piggyBac* experiment.

A schematic diagram of *pac*-puromycin *in vitro* selection procedures is shown in Supplementary Figure S3.

### Mutant ratio calculation by microscopy

About 10 μl of infected blood were collected in 200 μl of PBS, and 2 μl each of heparin solution and Hoechst33342 (1 mM) were added. The sample was then incubated at 37 °C for 5 min. After incubation, the sample was harvested at 500 × *g* for 5 min, and resuspended in 25–30 μl of RPMI1640. The suspension was placed on a glass slide, and the number of eGFP-positive parasites stained with Hoechst33342 was determined using fluorescent microscopy. The parasites were distinguished from white blood cells by fluorescence intensity and morphology of nuclei stained with Hoechst. At least 1,000 Hoechst-positive parasites were counted in several fields under 400× magnification. The mutant ratio was calculated by dividing the number of eGFP-positive parasites by the number of Hoechst-positive parasites. In experiments where transfection was performed with GFP-positive parasites, the mutant ratio was calculated by dividing the number of GFP-negative parasites by the number of Hoechst-positive parasites.

### Flow cytometry analysis

About 20 µl of infected tail blood (5 to 10% parasitemia) was collected in 1 ml of serum-free RPMI 1640 medium containing 1 µM of cell-permeant DNA Dye SYTO59 (Thermo Fisher Scientific) for counter staining, and incubated for 45 min at 20 °C^25^. The stained cells were analyzed with an EPICS ALTRA flow cytometer (Beckman Coulter, Brea, CA, USA) equipped with 488 nm argon lasers for GFP or eGFP, and 633 nm HeNe lasers for SYTO59. Parasites were gated using logarithmic forward/side scatter dot plots. The mutant ratio was analyzed by counting the number of GFP or eGFP-positive parasites among gated SYTO59-positive parasites. At least 19,000 SYTO59-positive parasites were analyzed. Data analysis was performed using program Kaluza ver. 1.5 (Beckman Coulter).

### Fluorescence analysis

Once parasite nuclei had been stained with Hoechst 33342, fluorescent images were obtained using a BZ-9000 fluorescence microscope (Keyence, Osaka, Japan) and were analyzed using a BZ-II Analyzer (Keyence).

### Drug sensitivity test

Wild type *P. berghei* were transfected with pXL/hdhfr-pac-egfp and EGF-pgT, selected using pyrimethamine *in vivo* ^23^, and named *HSP70-PAC*. Drug sensitivity tests were performed as previously described^6, 26^. Briefly, infected blood (parasitemia 1.0–3.0%) was resuspended in a culture medium to a final cell concentration of 2%. A 500-µl cell suspension was cultured with various concentrations of puromycin (0–15 µM) for 20 h in a 24-well plate. The parasites were harvested by centrifugation and analyzed using Giemsa-stained thin blood smear films. The number of mature schizonts per 300 parasites was counted and normalized to the control. Three experiments were performed in triplicate. IC_50_ values were calculated using program GraphPad Prism ver.5 (GraphPad, San Diego, CA, USA).

### Southern blot analysis

Two micrograms of extracted DNA were digested using *Eco* RI or *Eco* RV, separated on agarose gel, transferred onto a Hybond N^+^ membrane (GE Healthcare, Chalfont St. Giles, UK), and then hybridized with probes labeled using an AlkPhos Direct Kit (GE Healthcare). The signal was detected using a CDP-star detection reagent (GE Healthcare).

### Identification of *piggyBac* insertion site

In summary, genomic DNA of mutant parasites was digested using *Nde* I and then ligated with T4 DNA ligase. Inverse PCRs were performed using the primer F: ATGTCCAGGAGGAGAAAGGC, R: GCCCCCAAATAAAAACTTCC. The sequences of these products were obtained using an ABI3100 Analyzer, and the insertion sites were identified using the PlasmoDB database.

### Statistical analysis

Statistical analyses comparing each *pac*-integrated parasite clone against the wild type parasite were performed using two-tailed unpaired *t*-tests. The mutant ratio of each selection was compared using paired *t*-tests. IC_50_ values were calculated using program GraphPad Prism ver.5 (GraphPad).

## Electronic supplementary material

Supplementary information
