# Supplementary material for: High efficacy in vitro selection procedure for generating transgenic parasites of Plasmodium berghei using an antibiotic toxic to rodent hosts
Source: Sci Rep. 2017 Jun 21;7:4001. doi: 10.1038/s41598-017-04244-0 (PMC5479828; doi:10.1038/s41598-017-04244-0)
Supplement: Supplementary file 1 — Supplementary information [file 41598_2017_4244_MOESM1_ESM.pdf]

Supplementary Information

Title:

High efficacy *in vitro* selection procedure for generating transgenic parasites of *Plasmodium berghei* using an antibiotic toxic to rodent hosts

Authors:

Akira Soga[1], Hironori Bando[2], Mami Ko-ketsu[1], Hirono Masuda-Suganuma[1], Shin-ichiro Kawazu[1], Shinya Fukumoto[1]*

1. National Research Center for Protozoan Diseases, Obihiro University of Agriculture and Veterinary Medicine, Inada-cho, Obihiro, Hokkaido 080-8555, Japan
2. Department of immunoparasitology, Research Institute for Microbial Disease, Osaka University, Yamada-oka, Suita, Osaka 565-0871, Japan

*Corresponding author: Shinya Fukumoto (E-mail: fukumoto@obihiro.ac.jp)

Supplementary Figure S1. Full-length blot image used for Figure 2d.

WT: wild type, cl.: clone

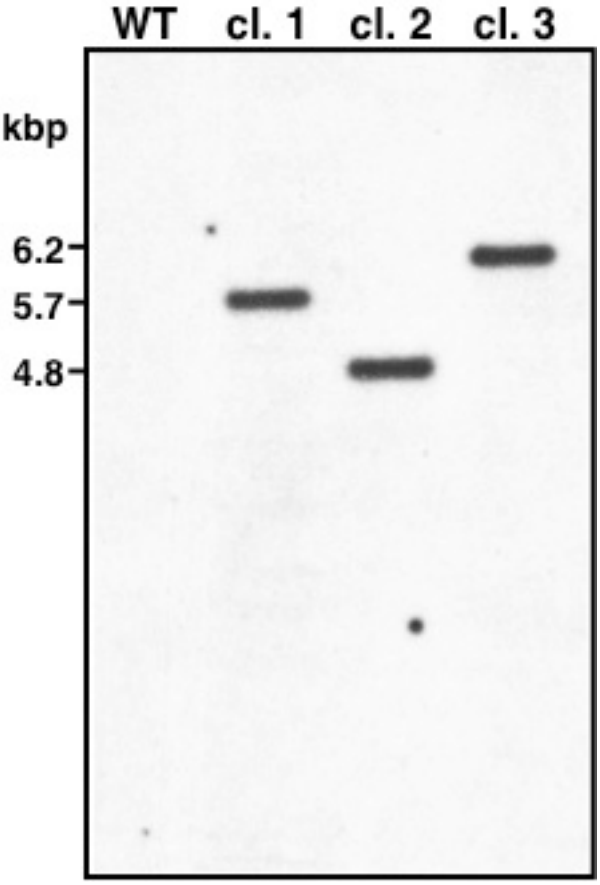

Supplementary Figure S2. Full-length blot images used for Figure 3c.

WT: wild type, GFP: GFP-expressing parasite (parental line), cl.: clone

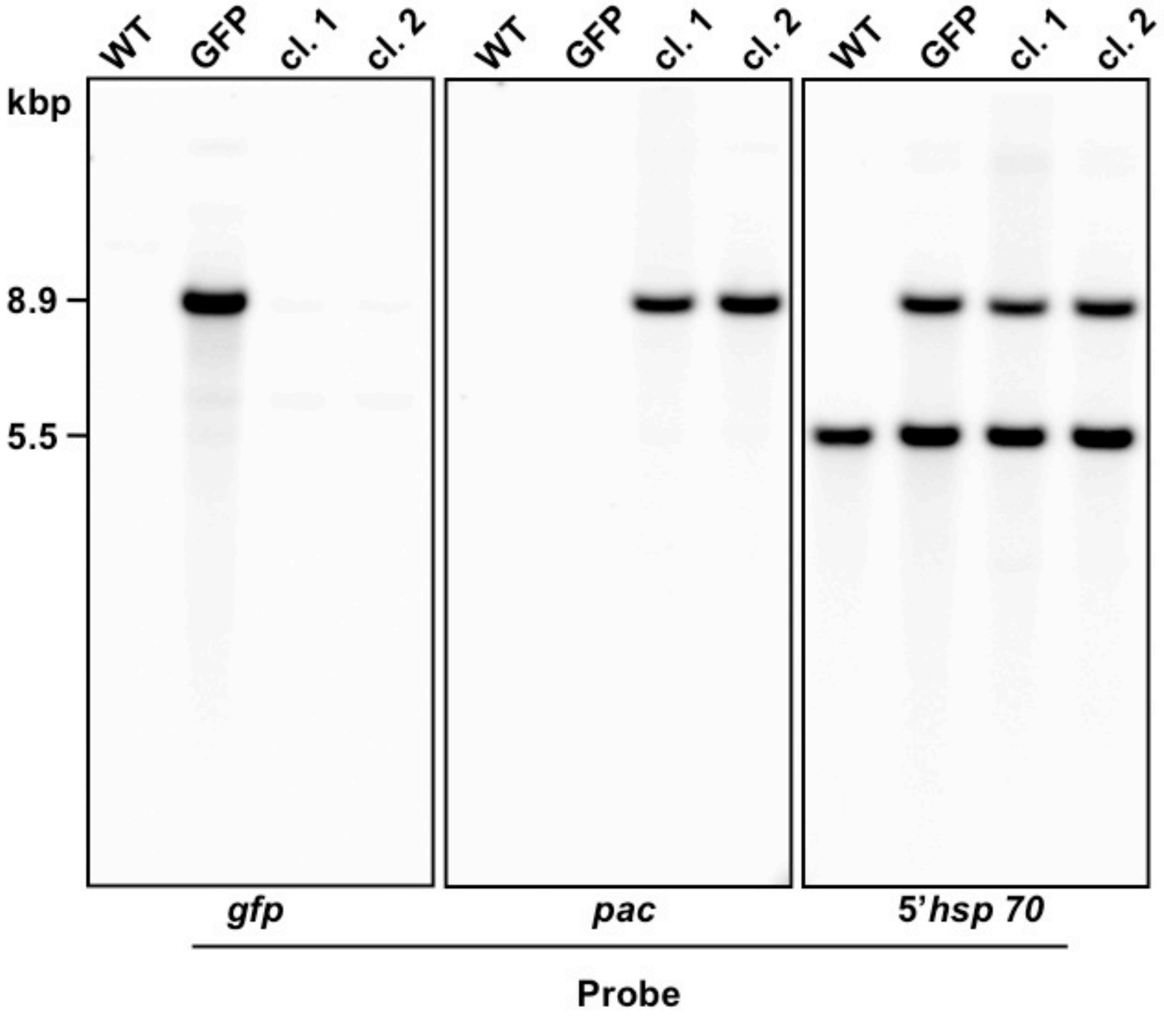

Supplementary Figure S3. Schematic diagram of *in vitro pac*-puromycin selection system.

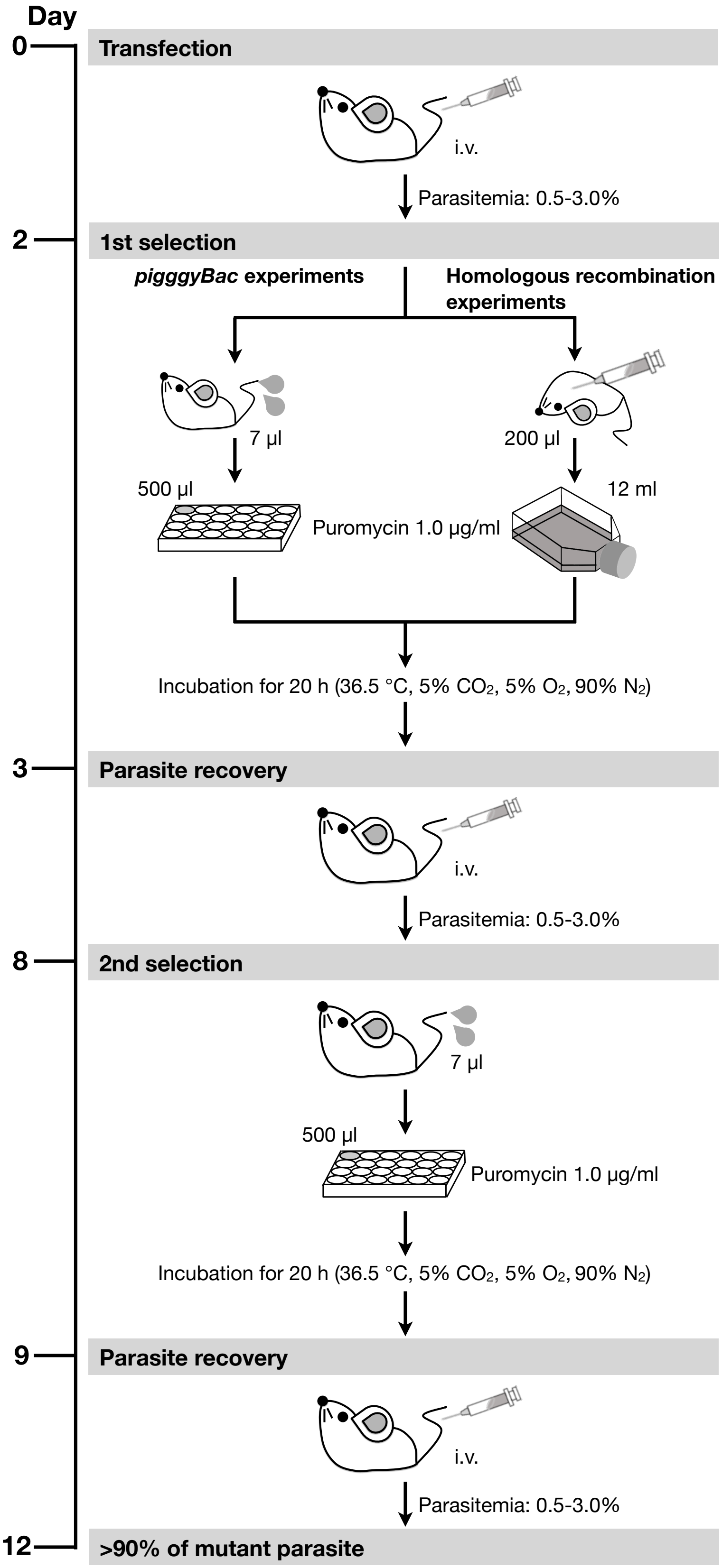

Table S1. Primer sequences used in Fig 4.

Primer name	Sequence (5’ to 3’)
P1	ACCCGTCTTTGGTCATTTGT
P2	AACAGCGCAGTTGAGTTGTAG
P3	TGTGCATATTATTTGTCATTTTTATG
P4	GGCTTGTACTCGGTCATGGT
P5	CTCGAGAGATCCCGTTTTTC
P6	TGGCCATTAAATCCACCATT
5’spect2-F	CGGATCCGCTAACACATAGCGAAACCATGTTGTC
5’spect2-R	CGCGAATTCTATAATCGTCATAATCATCTTCATCATCACC
3’spect2-F	CCGCTCGAGAAAGATGAAGAACAAAATGAGCATATAGATA
3’spect2-R	CGGTACCGCCAATTGTGTATTTTATGCAGTTTGACT
